# New Logic-In-Memory Paradigms: An Architectural and Technological Perspective

**DOI:** 10.3390/mi10060368

**Published:** 2019-05-31

**Authors:** Giulia Santoro, Giovanna Turvani, Mariagrazia Graziano

**Affiliations:** Dipartimento di Elettronica e Telecomunicazioni, Politecnico di Torino; Corso Castelfidardo 39, 10129 Torino, Italy; giovanna.turvani@polito.it (G.T.); mariagrazia.graziano@polito.it (M.G.)

**Keywords:** in-memory computing, logic-in-memory, non-von Neumann architecture, configurable logic-in-memory architecture, memory wall, convolutional neural networks, emerging technologies, perpendicular Nano Magnetic Logic (pNML)

## Abstract

Processing systems are in continuous evolution thanks to the constant technological advancement and architectural progress. Over the years, computing systems have become more and more powerful, providing support for applications, such as Machine Learning, that require high computational power. However, the growing complexity of modern computing units and applications has had a strong impact on power consumption. In addition, the memory plays a key role on the overall power consumption of the system, especially when considering data-intensive applications. These applications, in fact, require a lot of data movement between the memory and the computing unit. The consequence is twofold: Memory accesses are expensive in terms of energy and a lot of time is wasted in accessing the memory, rather than processing, because of the performance gap that exists between memories and processing units. This gap is known as the memory wall or the von Neumann bottleneck and is due to the different rate of progress between complementary metal–oxide semiconductor (CMOS) technology and memories. However, CMOS scaling is also reaching a limit where it would not be possible to make further progress. This work addresses all these problems from an architectural and technological point of view by: (1) Proposing a novel Configurable Logic-in-Memory Architecture that exploits the in-memory computing paradigm to reduce the memory wall problem while also providing high performance thanks to its flexibility and parallelism; (2) exploring a non-CMOS technology as possible candidate technology for the Logic-in-Memory paradigm.

## 1. Introduction

The von Neumann paradigm is the foundation of all modern computing systems. This paradigm is based on the exchange of data between a Central Processing Unit (CPU) and a memory. In particular, the CPU executes instructions on data that it retrieves from the memory, and writes back results in the memory. This data exchange mechanism is exacerbated when dealing with applications that require the manipulation of large data quantities (i.e., data-intensive applications). While through the years CPUs have become more and more powerful thanks to complementary metal–oxide semiconductor (CMOS) technology scaling, memories have not improved at the same rate, with the bandwidth being the main limitation. As a consequence, memories are not able to provide data as fast as CPUs are able to compute them. This problem is known as *von Neumann bottleneck* or *memory wall* and it limits the performance of systems based on the von Neumann architectural model as a lot of time is spent in retrieving data from the memory rather than computing them. This side effect is particularly visible when dealing with memory bound algorithms. Another critical consequence of the physical separation between the processing unit and the memory is related to the energy spent in performing memory accesses. In fact, especially for data-intensive applications, the large quantity of memory accesses required has a big impact on the overall power consumption. The very well known Moore’s law, according to which the number of transistors in an integrated circuit doubles every two years, has been obeyed for decades, but the growth rate predicted by Moore is now slowing down because of the limitations that technological scaling is facing. In fact, as foretold in the 2013 International Technology Roadmap for Semiconductors (ITRS) [1], CMOS scaling is reaching a boundary where further progresses will be impeded by physical, technological and economical limitations.

The drawbacks related to the von Neumann computing model and to the CMOS technology scaling are the main factors that drive this research. On the one side, the in-memory computational paradigm is explored as an alternative to the von Neumann one. The aim is to go beyond the conventional separation between computation and storage by integrating simple logic directly inside the memory cell. We refer to this approach as Logic-in-Memory (LiM). Its key benefits are mainly: (1) Bringing the computation directly inside the memory allows one to exploit the full internal bandwidth, mitigating the memory wall problem; (2) data are computed directly inside the memory without the need to move them between the computing and the storage units, drastically reducing the amount of memory accesses and the associated energy consumption and latency. On the other side, from a technological point of view, a non-CMOS technology, namely perpendicular Nano Magnetic Logic (pNML), is considered as a possible alternative to CMOS for implementing in-memory computing architectures as it intrinsically provides non volatility and computing capabilities in the same device.

The rest of this paper is organized as follows: Section 2 presents a taxonomy of the main in-memory computing approaches, based on how the memory is used for data computation; following the proposed taxonomy, we classify the main works found in literature. In Section 3 we present the main concepts and ideas behind the Configurable Logic-in-Memory Architecture (CLiMA) that is presented here for the first time. Section 4 describes an adaptation of CLiMA for quantized Convolutional Neural Networks that in Section 5 is compared to a non in-memory architecture and Section 6 describes the adaptation for pNML.

## 2. State of the Art

**Figure 1:**
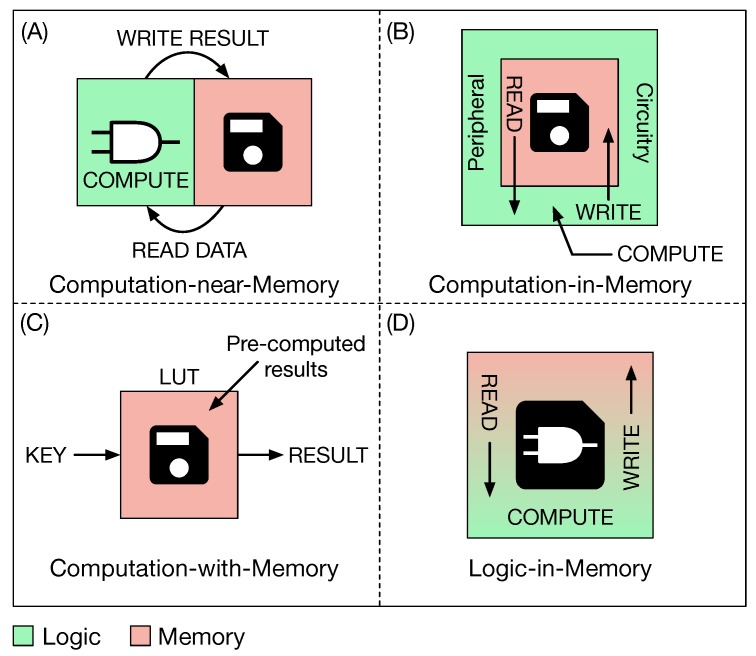
Depending on how the memory is used for computing data, four main in-memory computing approaches can be defined. (**A**) Computation-near-Memory (CnM): 3D-integration technologies allow one to bring computation and storage closer together by reducing the length of the interconnections. Logic and storage are still two separate entities. (**B**) Computation-in-Memory (CiM): The standard memory structure is not modified, while data computation is performed in the peripheral circuitry. (**C**) Computation-with-Memory (CwM): Memory is used as a Look Up Table to retrieve pre-computed results. (**D**) Logic-in-Memory (LiM): Data computation is performed directly inside the memory by adding simple logic in each memory cell.

The state of the art on in-memory computing is vast. The works found in literature differentiate from each other mainly for the role that the memory has in computing data. Starting from this observation, a taxonomy for classifying previous works was defined. According to this taxonomy the in-memory computing approaches can be divided in four main categories, as represented in Figure 1.

The four main approaches are described in the following.

(A) Computation-near-Memory (CnM, Figure 1A): Thanks to the 3D Stacked Integrated Circuit technology (3D-SIC) [2], computation and storage are brought closer together, from which the name CnM, by stacking the two units one on top of the other. This technique has a two-fold advantage: Reducing the length of the interconnections and widening the memory bandwidth. However, this approach cannot be considered as true in-memory computing, since computation and storage are still two separate entities, but more as an evolution of conventional architectures based on the von Neumann model. Works belonging to this category are [3,4,5,6,7,8].
(B) Computation-in-Memory (CiM, Figure 1B): The structure of the memory array is not modified, while its intrinsic analog functionality is exploited to perform computation. In particular, in-memory computation is achieved by reading data from the memory which is then sensed by sense amplifiers (SAs). SAs are specifically modified in order to support the computation of a few simple logic operations (AND, OR, ...). The result is then written back in the memory array. Decoders are also adapted in order to read more than one data from the array and execute row-wise (between data on different rows) or column-wise (between data on different columns) operations. Works belonging to this class are [9,10,11,12,13,14] and they all use a resistive non-volatile memory technology (RRAM). The approach followed in [15] is the same but here authors use a commodity volatile memory (DRAM, Dynamic Random Access Memory).
(C) Computation-with-Memory (CwM, Figure 1C): This approach uses memory as a Content Addressable Memory (CAM) to retrieve pre-computed results by means of a Look Up Table (LUT). The working principle of this kind of computation is that any Boolean function involving two or more inputs can be encoded in a memory by storing its truth table. In particular, input combinations are stored in a LUT, while results are stored in a CAM. Then the LUT is accessed through an input combination and an address is retrieved. These addresses are used to access the CAM and obtain the final result. Works that follows this approach are [16,17,18,19,20].
(D) Logic-in-Memory (LiM, Figure 1D): In this case logic is directly integrated inside the memory cell. Differently from the other three approaches, here data are computed locally without the need to move them outside the array (towards a close computing unit as in a CnM approach or towards the peripheral circuitry as in a CiM approach). Internal readings are performed in order to execute operations on data stored in different cells, by exploiting inter-cells connections. Internal writings are executed to locally save the result of the operation. There are a few works belonging to this category, such as [21,22,23,24].

## 3. Configurable Logic-In-Memory Architecture (CLiMA): Main Ideas

Our approach to in-memory computing, while mainly targeting the Logic-in-Memory concept, is not limited to it and also exploits the other approaches when required.

The novelties that we introduce with respect to existing works are manifold:

- The idea of an architecture that exploits various approaches to in-memory computing in order to adapt to different requirements and applications (Section 3);
- Configurability, hence flexibility, at different levels:
  - The basic block of CLiMA is a 1-bit Configurable LiM (CLiM) cell that can be programmed to perform different logic and arithmetic operations (Section 4.4);
  - More 1-bit CLiM cells can be grouped together to from a multi-bit CLiM cell that supports more complex operations such as bit-wise logic operations, multi-bit addition/subtraction, multiplication, shifts (Section 3 and Section 4.4);
- A data flow for Convolutional Neural Networks workload and an inter-cells connection fabric specifically optimized to minimize memory accesses outside CLiMA, to maximize data-reuse inside the CLiM array and to support high parallelism (Section 4.3, Section 4.4 and Section 4.5);
- A pNML-based design of the 1-bit and multi-bit CLiM cells and a small version of the CLiM array (Section 6).

We demonstrate the effectiveness of our approach by comparing CLiMA to a non in-memory Deep Learning Accelerator, showing promising results in terms of performance and a significant reduction of external memory accesses, which are the main limitations of the von Neumann bottleneck. The innovations presented in this work will be thoroughly explained and highlighted in the following sections.

### 3.1. Overview

**Figure 2:**
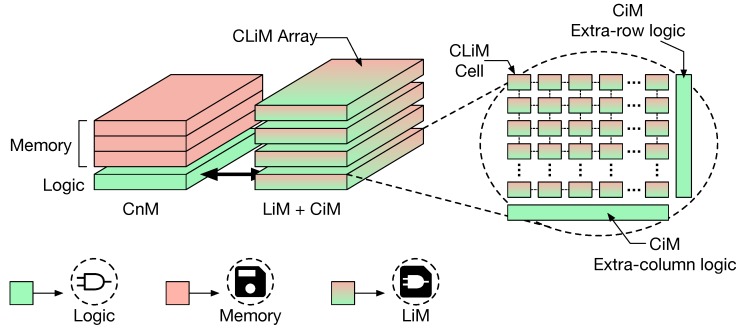
Conceptual structure of Configurable Logic-in-Memory Architecture (CLiMA): It can be seen as an heterogeneous unit that exploits configurability and different degrees of in-memory computation (CnM, CiM, LiM) to guarantee flexibility.

Figure 2 depicts the conceptual structure, in its most generic form, of the proposed in-memory computing architecture called CLiMA, Configurable Logic-in-Memory Architecture.

The key point in the definition of CLiMA is the flexibility. In fact, the idea is to conceive an architecture that well adapts to various applications that can benefit from in-memory computing in general and this means providing flexibility on different levels. In fact, applications differ for:

- Type of operations (logic, arithmetic);
- Complexity of operations (e.g., a logic function with respect to division);
- Data movement.

These parameters have an influence on the hardware requirements of the architecture. Depending on the type of operations and on their complexity, some of them can be executed directly in memory while others cannot. For this reason, as shown in Figure 2, CLiMA is conceived as a heterogeneous architecture composed of an in-memory (LiM and/or CiM) computing unit, the CLiM arrays, and a near-memory (CnM) computing unit. Operations that can be executed in-memory are dispatched to CLiM arrays, while the ones that cannot be executed in memory are assigned to the CnM unit. Each CLiM array is composed of different CLiM cells and, eventually, some extra-array (extra-row or extra-column) logic. A CLiM cell is thought as composed of a storage cell enhanced with simple logic that can be configured to perform different types of operations, from which the name Configurable Logic-in-Memory (CLiM) cell. The extra-array logic might be needed for further data processing outside the array and it can be considered as the CiM unit of CLiMA. The flexibility of CLiMA derives from its configurability (possibility of executing operations that differ for type and complexity) and from the presence of various degrees of in-memory computation (CnM, CiM, LiM).

### 3.2. Type of Operations and Data Movement in CLiM Array

**Figure 3:**
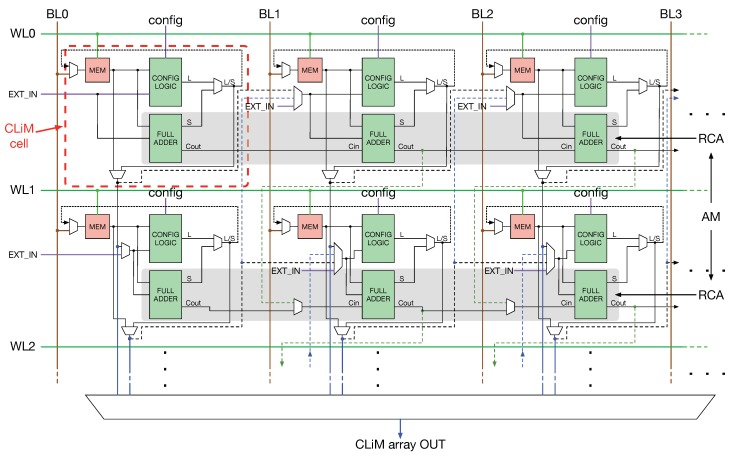
Detailed internal structure of the Configurable Logic-in-Memory (CLiM) array. Each CLiM cell can be represented as a logic-enhanced memory cell where data can be computed locally. By exploiting inter-cells connections it is possible to build more complex in-memory functions (e.g., a Ripple Carry Adder (RCA) or and Array Multiplier (AM)).

A more detailed view of CLiM array is shown in Figure 3.

The array is composed of CLiM cells whose reading/writing operations are controlled by bit lines (BL) and word lines (WL) as in a standard memory. Each CLiM cell is a logic-enhanced memory cell where data can be computed locally. In the example depicted in Figure 3, each CLiM cell is composed of a storage cell (MEM), a configurable logic block (CONFIG LOGIC) that can be configured to support different logic functions, and a full adder.

**Figure 4:**
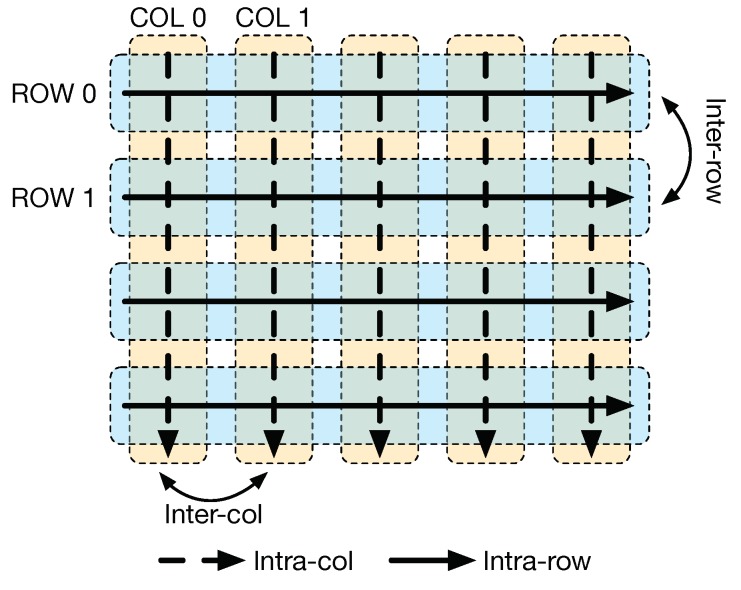
Possible types of data computation inside CLiM array.

In addition to the local data computation inside each cell, CLiM cells are interconnected between them in order to support other kinds of operations inside the array (Figure 4):

- Intra-row computation between cells in the same row (black dashed arrow in Figure 4);
- Intra-column computation between cells in the same column (black solid arrow in Figure 4);
- Inter-row computation between two rows, an instance being an operation between a data stored in row 0 and one stored in row 1;
- Inter-column computation between two columns, an instance being an operation between a data stored in column 0 and one stored in column 1.

Intra-row connections can be exploited to implement in-memory addition. In fact, as shown in Figure 3, full adders belonging to different cells can be connected together to propagate the carry and build a Ripple Carry Adder (RCA, highlighted by the red box). Similarly, inter-row connections can be used to build an Array Multiplier (AM) by connecting two RCAs. In this way, it is possible to implement complex arithmetic functions completely in memory. The disadvantage is that RCAs and AMs are not fast arithmetic circuits, hence, applications that have a large number of additions and/or multiplications might be slowed down (especially for what concerns multiplications, since an AM is much slower than a RCA). A solution to this problem could be to delegate these operations to a fast non in-memory unit when the considered application is characterized by a very large number of arithmetic operations.

## 4. CLiMA for Quantized Convolutional Neural Networks

On the basis of the ideas and concepts presented in Section 3, here a version of CLiMA is presented for quantized Convolutional Neural Networks. The reasons why CNNs have been chosen as target application are manifold:

- CNNs are an extremely popular application nowadays because they are a powerful method for solving many complex problems such as image recognition and classification, language processing, etc.;
- CNNs are data-intensive, hence, memory accesses represent the bottleneck;
- CNNs are computational-intensive, hence, they require hardware acceleration.

CLiMA is the ideal candidate for CNNs as it enables in-memory computation, drastically reducing the number of required memory accesses, and a high degree of parallelism, providing acceleration for time consuming applications like CNNs.

### 4.1. Convolutional Neural Networks (CNNs)

**Figure 5:**
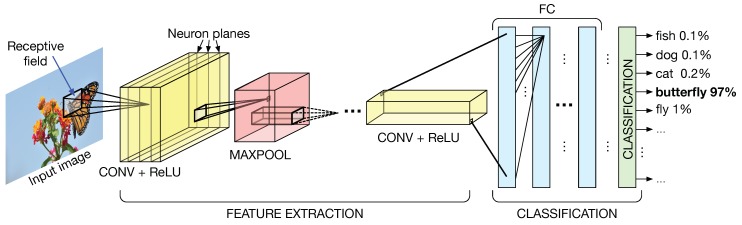
Convolutional Neural Networks (CNNs) are composed of different 3D layers. Each layer extracts different features from the input image.

Convolutional Neural Networks (CNNs) [25,26,27] are a family of Artificial Neural Networks used for pattern recognition and classification. A CNN, as depicted in Figure 5, is composed of many 3D layers that are responsible for feature extraction and classification.

Layers are three-dimensional as they are composed of a number of neuron planes, where each neuron analyzes a small portion of the input image, called the receptive field, extracting some key features. The feature extraction process is carried out by filtering the image with a kernel of weights (a filter), that is shared over a plane of neurons. The extraction of features by using the kernels of weights is called convolution, from which the name of the network. The output produced by the convolution operation is called the output feature map (i.e., the filtered image) and it is the input of the subsequent layer. Convolutional layers (CONV) are responsible for the extraction of features. Other type of layers are used to down-sample feature maps (e.g., maxpooling) or to introduce linear rectification (e.g., Rectifying Linear Unit (ReLU)). Fully connected (FC) layers are responsible for the actual classification.

**Figure 6:**
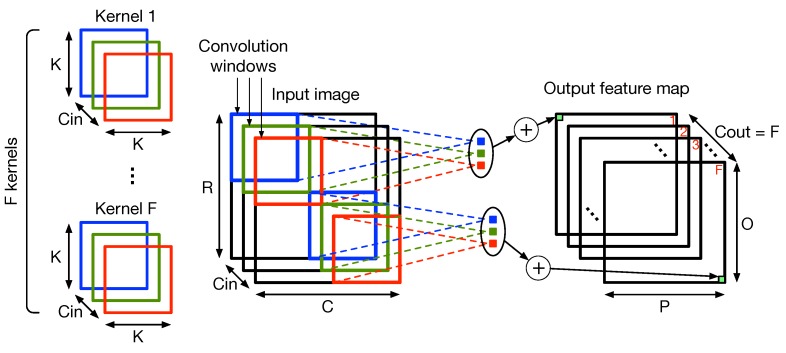
High-dimensional convolution operation.

Figure 6 shows in more detail how the convolution operation works.

The input image is usually composed of different input channels ($C_{in}$) with dimensions R×C. The kernels used to extract features have the same number of channels $C_{in}$ as the input image and dimensions K×K, which can vary in each layer. Kernels are slid on the input feature map by a quantity called stride (*S*). The number of kernels (*F*) determines the number of channels ($C_{out}$) of the resulting output feature map, which has dimensions O×P. The dimensions of the output feature map depend on the input image dimensions, the kernel dimensions and the stride, according to Equation (1).

(1) $$O=\frac{R-K}{S}+1;\;P=\frac{C-K}{S}+1.$$

CNNs are characterized by a complex structure and, over the years, network architectures have become more and more complex. The consequences of this growth are the need for very high-performance systems able to sustain such large throughput, and the increase of memory requirements because of the large number of parameters.

### 4.2. ShiftCNN: A Quantized CNN

Since an in-memory implementation can support only simple operations and limited precision, quantized CNNs are the perfect fit for in-memory computing architectures, since memory and computational requirements are greatly reduced in exchange for a small loss in prediction accuracy. In [28] authors propose to use power-of-two weights to eliminate the need for multiplications, which are instead transformed in simple shift operations. Moreover, according to their quantization algorithm, all weights are values of the type $2^{-n}$, hence, shift operations are all arithmetic right shifts. ShiftCNN has been chosen as target application for CLiMA.

### 4.3. CNN Data Flow Mapping Scheme for CLiMA

In this section we present a CNN data flow mapping scheme specifically optimized for CLiMA. Differently from the commonly used unrolling technique, this mapping scheme avoids data redundancy while guaranteeing parallel computation.

**Figure 7:**
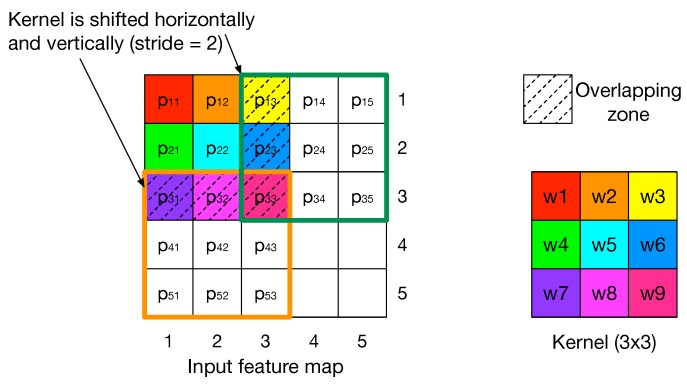
The kernel of weights is slid over the entire input image by a quantity called stride. The sub-region of the input image on which the kernel is applied is called convolution window. Convolution widows partially overlap.

The convolution operation, as highlighted in Figure 7, consists in applying a kernel of weights over the input feature map.

**Figure 8:**
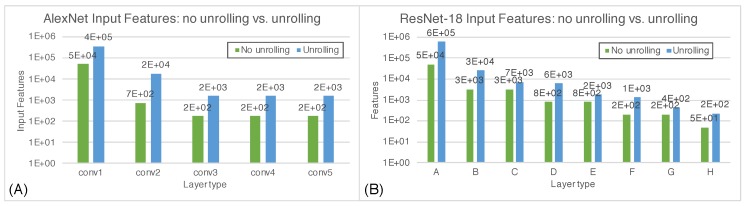
Data redundancy caused by unrolling in (**A**) AlexNet and (**B**) ResNet-18. Green columns represent the number of input features when applying no unrolling, blue columns represent the number of input features when applying unrolling. Input features are shown for each convolutional layer.

As explained in Section 4.1, the kernel is slid horizontally and vertically by a quantity called stride. In the example in Figure 7 the stride is equal to 2. The sub-region of the input feature map on which the kernel is applied is called the convolution window. It can be seen that convolution windows partially overlap so, in order to allow parallel computation, they are unrolled and overlapping regions are replicated causing data redundancy. The impact of unrolling convolution widows is exacerbated as the size of the kernel increases and the stride decreases, since the overlapping region gets larger. The graph in Figure 8 shows how the number of input features vary when applying unrolling, for each convolutional layer of two popular CNNs, AlexNet [29] and ResNet-18 [30].

**Figure 9:**
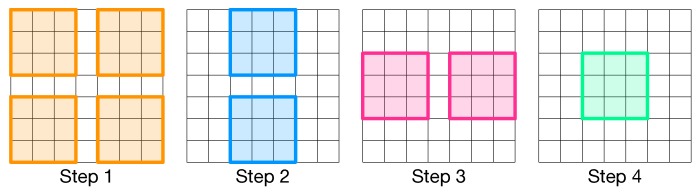
The convolution operation is divided in different steps. In each step only non-overlapping convolution windows are executed in parallel.

It can be seen that the data redundancy is not at all negligible as the number of unrolled input features (blue columns) increases of one order of magnitude with respect to the original number of features (green columns). For an architecture such as CLiMA, data redundancy is not acceptable since the storage space must be used in the most efficient way possible. For this reason, a different data flow mapping scheme is proposed. When executing convolution, not all convolution windows overlap, hence, those that do not overlap can be executed in parallel. As shown in Figure 9, the convolution operation can be divided in different steps in which only non-overlapping convolution windows are executed in parallel.

The number of steps to complete the convolution between a kernel of weights and an input feature map depends on the size of the input feature map, the kernel and on the stride. In the example in Figure 9, four steps are required to complete the convolution. The number of steps can be computed according to the following equation:

(2) $$\#steps=\frac{tot\_conv\_windows}{parallel\_conv\_windows}.$$

In Equation (2), tot_conv_windows is the total number of convolution windows while parallel_conv_windows is the number of non-overlapping convolution windows that can be executed in parallel. This number can be calculated as:

(3) $$parallel\_conv\_windows={\left(\frac{C}{K+(S-1)}\right)}^{2},\;K>1.$$

Equation (3) is valid for kernels with dimensions larger than one (K>1). When the kernel has size 1×1 the number of non-overlapping convolution windows is equal to the number of total windows.

It is clear that the advantage of this parallel non-overlapping data flow scheme is to avoid data redundancy while still guaranteeing parallel computation. This scheme can be naturally mapped on CLiMA by assigning a pixel of the input feature map to each cell of the array. Weights are instead properly distributed and shifted over the array (Section 4.5).

### 4.4. CLiM Array Structure

**Figure 10:**
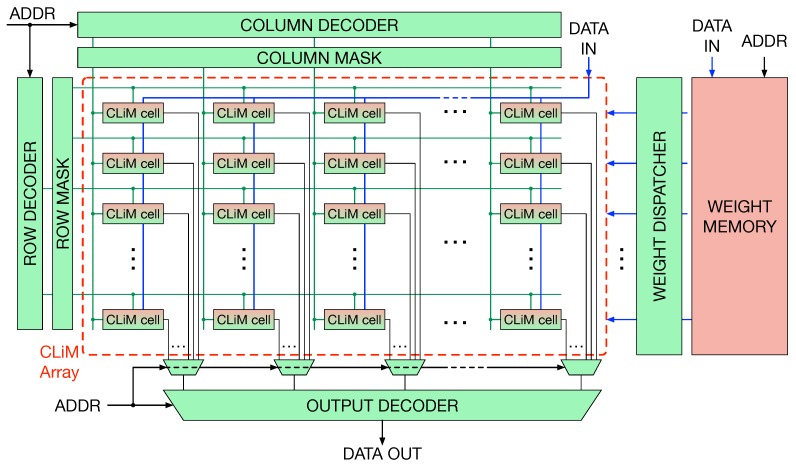
Architecture of CLiMA for quantized CNNs.

Figure 10 depicts the architecture of CLiMA for quantized CNNs.

**Figure 11:**
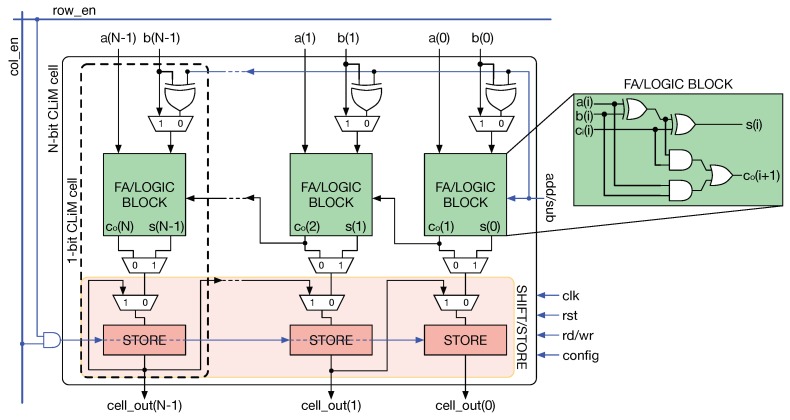
Internal structure of the CLiM cell. Many 1-bit CLiM cells are properly interconnected, exploiting inter-cell connections, to build a more complex N-bit CLiM cell.

The main core of CLiMA is the array of CLiM cells. Each CLiM cell has both storage and computation capabilities. Modified row and column decoders are used to control the data flow inside the array. Weights are read from a weight memory, external to the array, and dispatched through a weight dispatching mechanism. More details on decoders and the weight dispatcher will be given in Section 4.5. The internal structure of the CLiM cell is shown in Figure 11.

It can be seen that many 1-bit CLiM cells are properly interconnected, by exploiting inter-cell connections, to create a more complex N-bit CLiM cell. Each 1-bit cell is composed of a configurable computational block, a storage cell and other simple logic. The computational block is a Full Adder (FA) that can also be used to perform logic operations by fixing one or more of the FA inputs to logic 0 or 1, as shown in Table 1.

In order to support multi-bit addition, the output carry (C_out_) of the FA inside a 1-bit CLiM cell is connected to the input carry (C_in_) of the adjacent 1-bit cell. By exploiting inter-cell connections it is possible to build an in-memory Ripple Carry Adder (RCA). In addition, storage cells are interconnected in a chain-like manner in order to implement a multi-bit storage block that can also work as a shift register. Only right shifts are supported in the case represented in Figure 11 since, as explained in Section 4.2, ShiftCNN requires only those. Nonetheless, with very simple modifications left shifts can also be handled. Moreover, for the sake of clarity, Figure 11 does not show the presence of redundant storage blocks (one for each 1-bit cell, in addition to the one that is also used as the shift register). The redundant storage block is used to retain partial results that will be reused for further elaboration.

**Figure 12:**
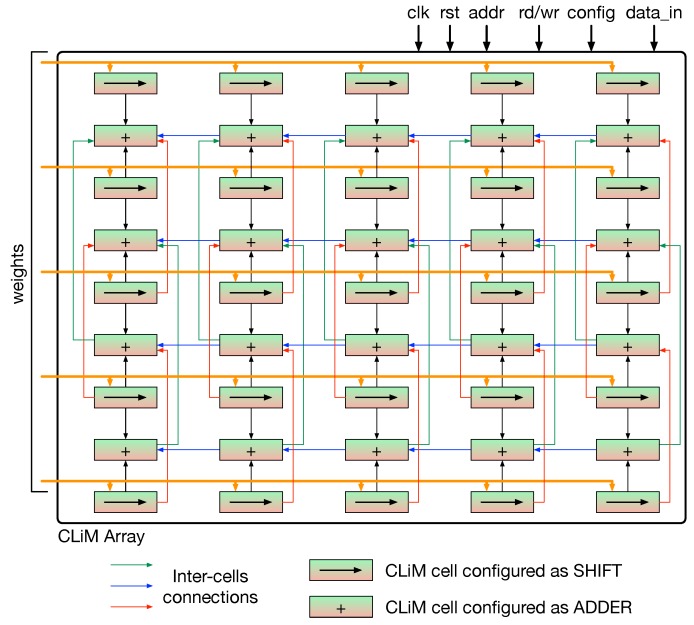
Interconnection fabric inside the CLiM array.

The architecture depicted in Figure 10 does not show the interconnections between CLiM cells. These interconnections have been specifically designed to support CNN-like data flow inside the array. A detailed scheme of the interconnection fabric inside the CLiM array is shown in Figure 12.

**Figure 13:**
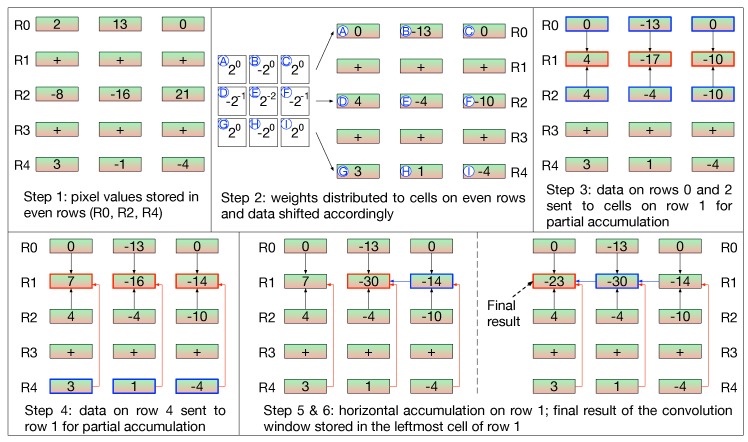
Management of convolution computation inside the CLiM array.

Furthermore, rows of CLiM cells are alternatively configured as shift registers (even rows) and adders (odd rows). The idea is to store pixels of the input feature map inside shift cells where they are also locally shifted according to the value of the correspondent weight. Then the shifted pixels are accumulated in the cells configured as adders. Figure 13 clarifies how convolution is managed inside the array.

In particular, the computation of a 3×3 convolution window is shown as example. The interconnection fabric has been designed to be flexible, hence, it can support any kernel size.

### 4.5. Weight Dispatching Mechanism

In order to support the parallel non-overlapping data flow scheme shown in Figure 9, weights must be properly dispatched to the cells inside the CLiM array. In order to do so, the combined action of the weight dispatcher and row/column decoders is exploited. Row/column decoders are modified in order to activate multiple adjacent rows/columns. A starting and an ending address are provided to decoders that will consequently activate all rows/columns comprises between the starting and the ending address. Since, as it can be notice from Figure 9, parallel convolution windows might not be adjacent, row/column masks are used to disable those rows or columns comprised between the starting and ending address which must remain inactive. The weight dispatcher is used to properly shuffle weights over the array.

**Figure 14:**
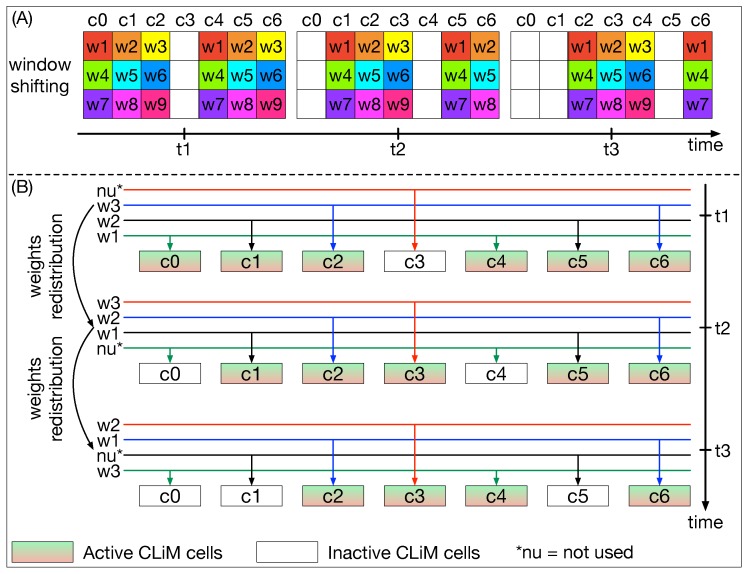
(**A**) Convolution windows are shifted over the array by properly activating/inactivating rows and columns. (**B**) The weight dispatcher properly distributes weights inside the CLiM array in order to reproduce the convolution window shifting process.

As highlighted in Figure 14A, the window shifting process is obtained by controlling which cells are active and which are not, step after step. At the same time, weights are properly shuffled, as shown in Figure 14B, so that they are distributed to the correct cells.

The weight dispatching mechanism has been optimized for 3×3 kernels since they are the most common ones. Nonetheless, other kernel sizes can be also supported.

### 4.6. Data Reuse Possibilities

One of the main reasons for exploiting a Logic-in-Memory architecture such as CLiMA for Convolutional Neural Networks is the possibility of reusing data already stored and computed inside the array for further processing, without any need to move it outside.

**Figure 15:**
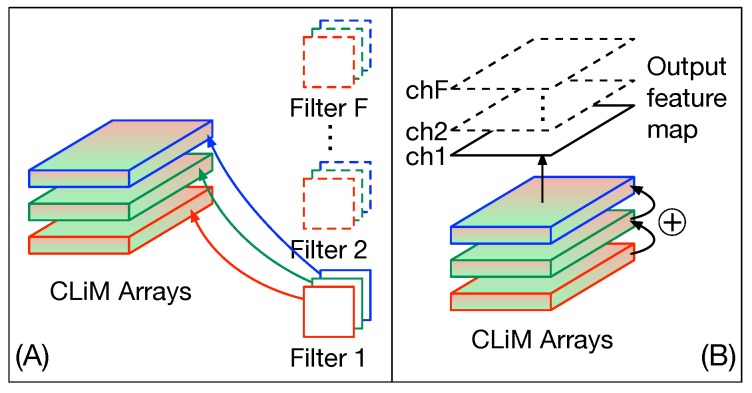
Data reuse in CLiMA. (**A**) Filters are reused across input feature maps according to the sliding window process. Input feature maps are also reused by different filters. (**B**) Partial results are reused for further processing to obtain the final output feature maps.

The possibilities for data reuse in CLiMA are summarized in Figure 15 and explained in the following.

- Filters are reused across input feature maps according to the sliding window process (Figure 15A).
- Input feature maps are reused by different filters (Figure 15A).
- Partial results are reused for further processing (cross-channel accumulation) to obtain the final output feature maps (Figure 15B).

## 5. Results and Discussion

Before bounding it to any technology, CLiMA was modelled by using a fully parametric VHDL (VHSIC Hardware Description Language) code that was validated by means of extensive simulations and by comparing the obtained results to those obtained from an analogous model developed in MATLAB. Moreover, in order to prove the effectiveness of the CLiMA computational model, it has been compared to a conventional (non in-memory) Deep Learning Processor presented in [31,32].

An analytic computational model of CLiMA was defined. This model takes into account the following parameters:

- Convolutional layer parameters including input feature map dimensions (R,C), kernel dimensions (*K*), stride (*S*) and output feature map dimensions (O,P);
- The number of parallel non overlapping convolution windows;
- The number of execution cycles needed to complete a convolution window.

The total number of convolution widows in a layer depends on the size of the output feature map, that is given by the following equation:

(4) $$O=P=\frac{R-K}{S}+1.$$

We are assuming that input and output feature maps and kernels are square, hence, they have the same width and height (R=C, O=P). The total number of convolution widows, $CW_{tot}$, is then equal to:

(5) $$CW_{tot}=O\;\cdot \;P=O^{2}=P^{2}.$$

The number of non overlapping convolution windows, $CW_{non-ov}$, is given by the following expression:

(6) $$CW_{non-ov}={\left(\frac{R}{K+(S-1)}\right)}^{2}.$$

According to the data flow mapping scheme presented in Section 4.3, a certain number of steps is needed to complete a convolution operation. This number, $C_{steps}$, is equal to the upper bound of the ratio between the total number of convolution windows $CW_{tot}$ and the number of parallel non-overlapping ones $CW_{non-ov}$:

(7) $$C_{steps}=\left\lceil \frac{CW_{tot}}{CW_{non-ov}}\right\rceil .$$

The number of execution cycles, $C_{cycles}$, needed to complete a full convolution operation on a layer is given by the product between the number of cycles to execute a single convolution window, $CW_{cycles}$, and $C_{steps}$:

(8) $$C_{cycles}=CW_{cycles}\;\cdot \;C_{steps}.$$

$CW_{cycles}$ depends on the size of the convolution window that, in turn, depends on the size of the kernel. Moreover, by taking into account how a convolution window is mapped and executed inside CLiMA, the term $CW_{cycles}$ can be calculated as following:

(9) $$CW_{cycles}=8+1+\left(\frac{K-1}{2}\right)+\left(K-1\right).$$

In Equation (9) the following factors are taken into account:

- The number of cycles to execute shift operations; in CLiMA data are shifted 1 bit at a time. Since weights are 8-bit long, in the worst case scenario eight cycles are needed to complete the operation;
- The number of cycles to execute accumulations:
  - One cycle for partial accumulation of data couples (Figure 13, step 3); this term does not depend on the size of the kernel because these accumulations can always be done in parallel;
  - (K−1)/2 cycles for partial accumulation of non-adjacent data (Figure 13, step 4); this term depends on the size of the kernel, in fact, as the convolution window dimension changes the number of non-adjacent data to accumulate changes as well;
  - K−1 cycles to perform final horizontal accumulations (Figure 13, steps 5 and 6); similarly to the previous term, also this one depends on the size of the kernel.

Equations (7) and (9) can be substituted in Equation (8) to obtain the total number of cycles required to execute a full convolution operation of a layer.

**Figure 16:**
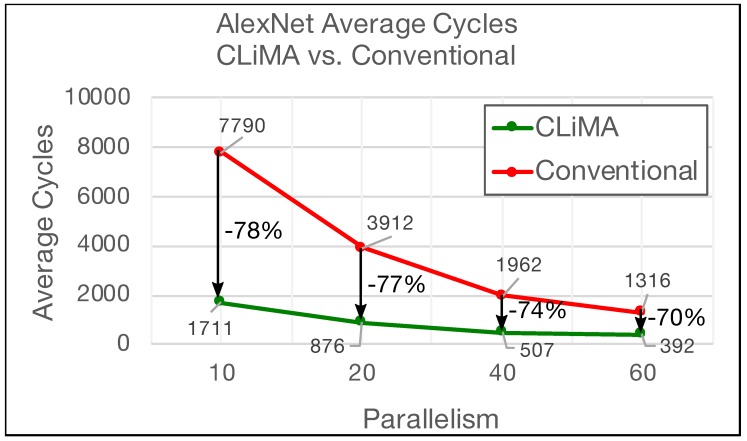
Average cycles needed to execute AlexNet in different scenarios: CLiMA vs. Conventional.

**Figure 17:**
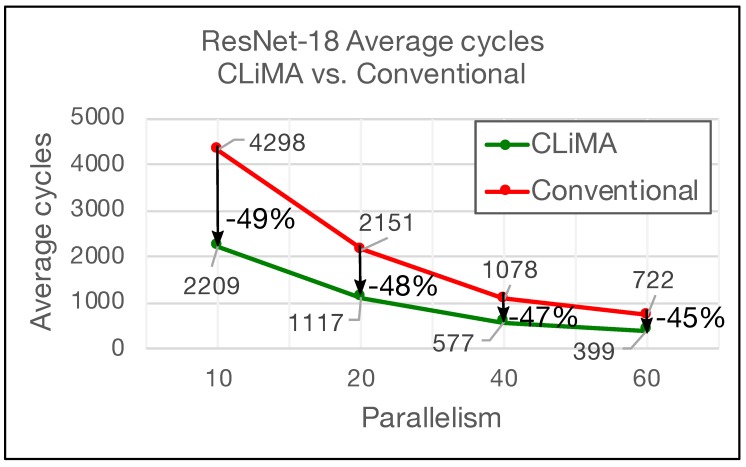
Average cycles needed to execute ResNet-18 in different scenarios: CLiMA vs. Conventional.

This simple but effective computational model was used to extract results and carry out comparisons between CLiMA and the Deep Learning Processor, by considering AlexNet and ResNet-18. The Deep Learning Processor is composed of a number of Processing Elements (PEs) that are capable of performing different types of operations including Multiply-Accumulate (MAC) ones. PEs work in parallel and each of them has a throughput of 1 MAC per cycle. Assuming that each PE executes a convolution window, it takes K×K cycles to complete a single convolution window. For what concerns CLiMA, the assumption is that a certain number of non-overlapping convolution windows is executed in parallel inside the array. In order to perform comparisons, four different scenarios were considered. The difference between these scenarios is the parallelism that, for the Deep Learning Processor, is referred to the number of parallel PEs, while for CLiMA, it is referred to the number of parallel non-overlapping windows. Figure 16 and Figure 17 report the average number of clock cycles needed to perform a complete convolution in different parallelism scenarios for AlexNet and ResNet, respectively.

The average number of clock cycles is simply calculated by averaging the number of clock cycles needed to complete the convolution of each layer in the considered CNN. In both graphs, the parallelism level is reported on the *x* axis, while the average number of clock cycles is shown in the *y* axis. It can be clearly seen that, for both the CNNs and for all the parallelism scenarios, CLiMA outperforms the Deep Learning Accelerator. In the AlexNet case, the average cycles are reduced by 78% percent in the worst parallelism scenarios (only 10 PEs or non-overlapping convolution windows). The percentage reduction slightly decreases as the parallelism increases, reaching −70% in the best parallelism scenario (60 PEs or non-overlapping convolution windows). For what concerns ResNet, the trend shown in Figure 17 is similar to the AlexNet one, except that the difference between the average cycles of CLiMA with respect to the Deep Learning Accelerator is smaller. In fact, it ranges from −49% in the worst parallelism scenario to −45% in the best.

For both the CNNs, CLiMA provides a reduction in terms of average cycles needed to complete the convolution in all the layers of the network that is higher when the parallelism level is smaller, as compared to the Deep Learning Accelerator, further proving the effectiveness of the CLiMA computational model. The reduction difference between AlexNet and ResNet-18 depends on the characteristics of the two networks (i.e., layers and kernels dimensions, number of channels etc.).

The VHDL code used to describe CLiMA was synthesized in order to get an estimation of the maximum working frequency at which the architecture can run. The technology used for the synthesis is the same used for the Deep Learning Accelerator and it is a commercial 28nm FDSOI (Fully Depleted Silicon-on-Insulator). For both architectures a parallelism of 10 has been chosen and the maximum reachable working frequency, in both cases, is approximately 1.8GHz. The working frequency was used to compute the execution time required by CLiMA and the Deep Learning Processor to run ALexNet and ResNet-18 when the parallelism is 10. Results are reported in Table 2.

When comparing the two architectures, since the working frequency is the same, whereas the number of average cycles required by CLiMA is much lower than what the Deep Learning Accelerator requires, the resulting execution time needed to complete the convolution of Alexnet and ResNet-18 is, respectively, 45× and 20× lower for CLiMA with respect to the Deep Learning Accelerator.

The main figure of comparison between the two architectures is related to the number of memory accesses. In fact, we want to demonstrate that not only is the CLiMA computational paradigm effective in terms of execution acceleration thanks to its intrinsic massive parallelism, but it is also effective in reducing the data exchange between the processing unit and the memory. When considering CLiMA, as shown in Figure 10, we can identify the computing core that is the CLiM array and an external memory that is the weight memory. This memory is accessed to retrieve the weights that are reused over all the convolution windows inside a feature map, therefore, requiring only K×K read operations. We are assuming that the input features are already stored inside each CLiM cell of the array, neglecting the write operations required to load them for the first time as this is an initialization operation that cannot be avoided. Once the convolution operation is completed, the final results, which are then reused for cross-channel accumulation, are already stored inside the CLiM array, hence, no further external write or read operation is needed.

When considering, instead, the Deep Learning Accelerator, both input features and weights are continuously read from an input buffer and passed to the execution unit that performs MAC operations and then writes results into an output buffer. Therefore, the number of read/write operations to input/output buffers, when considering all convolution windows in a layer, is:

- 2×(K×K)×tot_conv_windows read accesses to the input buffer to retrieve input features and weights;
- O×P write accesses to the output buffer to store the convolution results.

As for CLiMA, we are not considering that input features and weights must be loaded from an external memory into the input buffer because it is an unavoidable operation.

**Figure 18:**
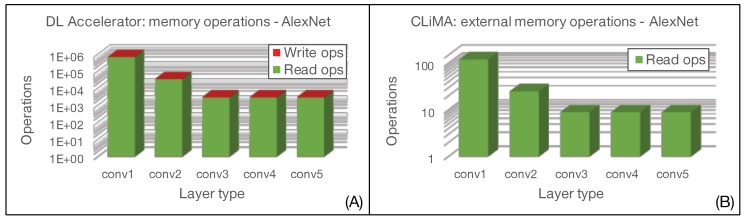
Memory access evaluation for AlexNet in (**A**) Deep Learning Accelerator and (**B**) CLiMA.

**Figure 19:**
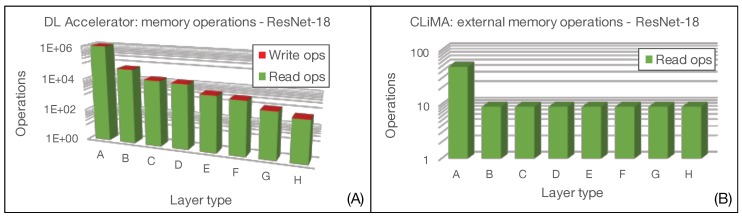
Memory access evaluation for ResNet-18 in (**A**) Deep Learning Accelerator and (**B**) CLiMA.

Figure 18 and Figure 19 show the comparison in terms of memory accesses between CLiMA and the Deep Learning Accelerator for AlexNet and ResNet-18, respectively. It can be clearly noticed that the in-memory computational model and the data reuse possibilities offered by CLiMA make it possible to drastically reduce the number of memory accesses with respect to a non-in-memory conventional paradigm, such as the one used in the Deep Learning Processor.

In general, comparing CLiMA to other architectures (either in-memory or conventional ones) is not easy because of architectural, technological and computational differences. As a result, the risk is that the comparison might be unfair. In addition, most of the time, papers lack of details about how the proposed architectures manage the computation or how there are no common comparison figures. This makes comparisons even more difficult and, for this reason, CLiMA was only compared to a conventional architecture (the Deep Learning Processor) about which we had sufficient details to be able to extract some useful data.

## 6. Beyond CMOS: A pNML Implementation

Perpendicular Nano Magnetic Logic (pNML) [33] is considered one of the most promising alternative technologies to CMOS [34] and it is perfect for in-memory computation as it intrinsically provides both non-volatility and computing capabilities [35,36]. In addition, pNML offers 3D integrability and low power consumption, all characteristics that make this technology ideal for overcoming the issues related to von Neumann architectures and CMOS scaling.

### 6.1. pNML Basics

**Figure 20:**
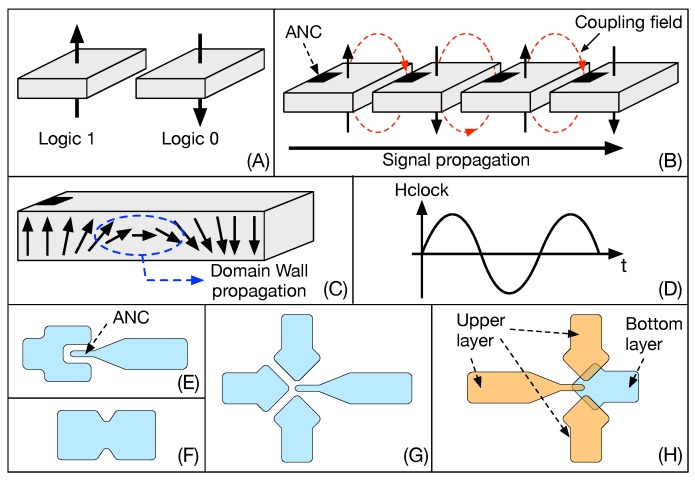
pNML basics. (**A**) The magnetization direction encodes logic ‘0’ and ‘1’. (**B**) The Artificial Nucleation Center (ANC) guarantees correct signal propagation in a perpendicular Nano Magnetic Logic (pNML) chain of magnets. (**C**) Domain wall propagation inside the nanomagnet causes the switch of the magnetization direction. (**D**) Global out-of-plane magnetic field used as clocking mechanism. (**E**) Inverter. (**F**) Notch. (**G**) Minority voter. (**H**) 3D minority voter.

pNML is based on the nanomagnet, a small (∼tens of nanometers) single domain multi-layer Co/Pt device that has bi-stable behavior. This means that, because of the perpendicular (from which the name perpendicular NML) magnetization anisotropy, it can be only in two stable magnetization states that depend on the direction of the magnetization. These states can be used to encode logic ‘0’ and logic ‘1’, as shown in Figure 20A.

Signal propagation in pNML depends on the magneto-static field-coupling interactions between nanomagnets [37]. In order to propagate the information in a specific direction, the magnetic properties of a small region of the nanomagnet are modified through Focused Ion Beam (FIB) irradiation [38]. The irradiated region is called the Artificial Nucleation Center (ANC). As shown in Figure 20B, neighboring pNML cells couple in a parallel or anti-parallel way, depending on their relative position, favoring signal propagation in a direction that depends on the position of the ANC. The ANC is the point where the nucleation of a domain wall starts and eventually propagates inside the magnetic device (Figure 20C). ANCs can also be obtained by changing the shape and thickness of the nanomagnet [39] (Figure 20E). The propagation of information inside pNML circuits is obtained thanks to an external magnetic field (sinusoidal as shown in Figure 20D) that is applied globally to the circuit [40]. This external magnetic field has the same function of the clock signal in CMOS circuits. Thanks to the combined action of ANCs and the clocking field, information propagation can be correctly controlled in pNML circuits. The elementary pNML blocks with which any logic circuit can be built are the inverter (Figure 20E), the notch (Figure 20F) and the minority voter (Figure 20G and 3D version in Figure 20H). The notch works as a barrier, blocking the signal propagation unless a short depinning magnetic field is applied [41]. This block can be used to create memory elements [42,43]. Moreover, pNML technology allows one to build 3D structures by stacking different layers of nanomagnets [44,45,46,47]. Previous works such as [42,48,49,50,51,52] already explore the potentialities of NanoMagnetic Logic architectures (3D and non), but none of them propose a complete Logic-in-Memory design, which is instead presented in the following.

### 6.2. pNML-Based CLiM Array

**Figure 21:**
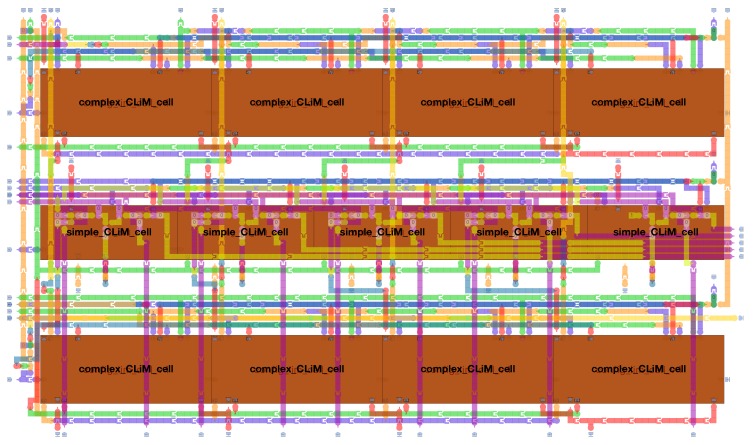
Small pNML-based version of the CLiM array.

Figure 21 depicts a small pNML-based version of the CLiM array described in Section 4.4.

**Figure 22:**
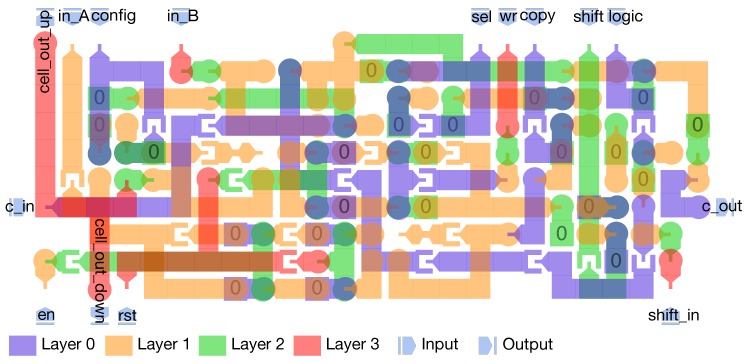
Complex pNML cell.

**Figure 23:**
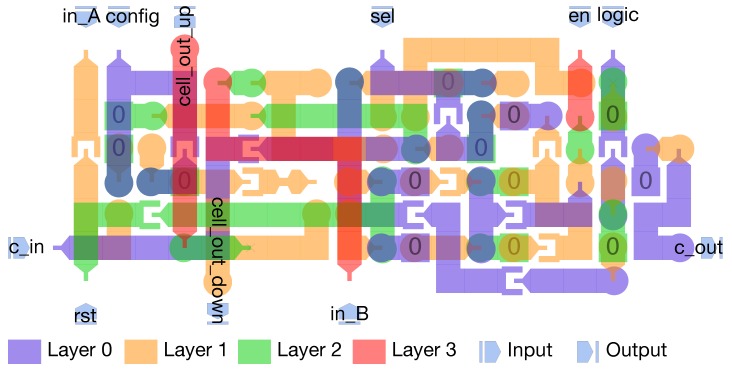
Simple pNML cell.

The design of the array was done by using MagCAD (https://topolinano.polito.it) [53], a CAD for emerging technologies developed at the VLSI Laboratory (research group in the Department of Electronics and Telecommunication Engineering of Politecnico di Torino). MagCAD allows one to design pNML structures thanks to an intuitive and simple GUI (Graphical User Interface) in which elementary blocks can be combined together to form more complex structures and 3D designs. Starting from the designed structure, MagCAD allows the extraction of the VHDL description of the circuit, that is based on a compact VHDL model [35] of pNML devices. The generated VHDL can be used to simulate (using a common HDL simulator) and verify the functionality of the circuit [54,55,56]. The complexity of the pNML-based CLiM array depends on the complexity of the interconnections between CLiM cells, as it can be noticed from Section 4.4. This strongly limits the size of the design that can be implemented by hand, without any support for the routing. The design in Figure 21 uses nine layers of nanomagnets. There are two types of cells used for the pNML array, one called complex (Figure 22) and the other the simple (Figure 23) CLiM cell. Both are based on the structure shown in Figure 11, the only difference between them being that the simple CLiM cell does not support shift operations and does not have the redundant storage block. The simple CLiM cell can be used in the odd rows of the array that perform only accumulations.

Both the cells have four layers of magnets. Based on the dimensions of the nanomagnets, that in these designs are 30×50nm, the area occupied by the complex cell is 22.5μm, while the simple cell occupies 14.4μm. The area of the CLiM array is 582μm and interconnections occupy a big portion of it because of their complexity.

Even though in the designs here presented we have used relatively large nanomagnets (30×50nm), pNML can be easily scaled to improve compactness. The designs could be also improved in order to reduce the impact of interconnections on the overall area occupation, however, as already said, the lack of an automatic and optimized routing tool makes it challenging. Nonetheless, the non-volatile nature of the technology and the total absence of current flow and leakage sources makes it an ideal beyond-CMOS technology for in-memory computing.

## 7. Conclusions

The Configurable Logic-in-Memory architecture that we have presented has strong points and issues that are worth being analyzed. Regarding its advantages, CLiMA provides:

- In-memory computation: Data are processed directly inside the memory, drastically reducing the need of data movement and favoring their reusing for further computation;
- Parallelism: The array is intrinsically highly parallel and perfect for accelerating compute and data intensive applications;
- Flexibility: The configurability of the cells and the possibility of exploiting inter-cells connections to build complex in-memory functions make CLiMA adaptable to different applications.

Regarding its limitations, mainly two can be identified:

- Not all data-flows can be supported in an array-like structure because moving data from any source to any destination is not easy and would require a very complex (but flexible) network of interconnections;
- The control of data movement between cells is complex and must be managed carefully in order to avoid cells receiving/sending wrong data from/to wrong cells.

To conclude, the Logic-in-Memory paradigm seems to be a promising alternative to the von Neumann one. We have defined a novel Configurable Logic-in-Memory Architecture that relies on in-memory computation, flexibility and parallelism to tackle the memory bottleneck problem while also providing high performance.

## Figures and Tables

**Table 1:** Logic operations that can be performed with a Full Adder by fixing one or more of the inputs. In this case A, B and C_in_ are the three inputs while S and C_out_ are the output (sum and output carry,  respectively).

Fixed Input	S	C_out_
A = 0	B ⊕ C_in_	B · C_in_
A = 1	$\bar{B⊕{\text{C}}_{\mathrm{in}}}$	B + C_in_
A = 0 & B = 1	$\bar{{\text{C}}_{\mathrm{in}}}$	C_in_
A = 1 & B = 0	$\bar{{\text{C}}_{\mathrm{in}}}$	C_in_

**Table 2:** Performance estimation of CLiMA with respect to the Deep Learning Accelerator for AlexNet and ResNet-18 when the parallelism is 10. For both architectures the working frequency is 1.8GHz.

CNN Type	Architecture	Average Cycles	T_exec_ (μs)
AlexNet	CLiMA DL Acc.	1711 7790	0.95 43.2
ResNet-18	CLiMA DL Acc.	2209 42,939	1.2 24
